# A systematic review investigating prenatal cannabis and tobacco co-exposure: Impacts on neonatal, behavioral, cognitive and physiological outcomes

**DOI:** 10.1016/j.dadr.2025.100376

**Published:** 2025-08-29

**Authors:** Mathilde Argote, Leah Hilson, Maryam Sorkhou, Rachel A. Rabin

**Affiliations:** a McGill University, Montreal, Canada; b The Douglas Mental Health University Institute, Verdun, Canada; c Institute of Medical Sciences, University of Toronto and Institute for Mental Health Policy Research, Centre for Addiction and Mental Health, Toronto, ON, Canada; d Department of Psychiatry, McGill University, Montreal, Canada

**Keywords:** Cannabis, Tobacco, Cannabis and Tobacco co-use, Nicotine, Prenatal offspring

## Abstract

**Background:**

Despite the high and increasing rates of cannabis and nicotine/tobacco product (NTP) use during pregnancy, the impact of their combined use on health outcomes in offspring remains poorly understood. Given the growing body of research on prenatal cannabis and NTP co-exposure and its effects on neonatal, behavioral, cognitive, and physiological outcomes in offspring, we conducted a systematic review to synthesize the existing literature and evaluate whether prenatal co-exposure results in additive and/or synergistic adverse effects compared to prenatal cannabis-only exposure and prenatal NTP-only exposure.

**Methods:**

We searched Medline, Embase, and PsycINFO databases via OVID for human and animal studies examining the association between prenatal co-exposure and single-substance exposure on neonatal, behavioral, cognitive, and physiological outcomes in offspring.

**Results:**

Of 3217 records identified, 46 articles were included in the review (human, n = 43; preclinical n = 3). For select neonatal outcomes, co-exposed infants exhibited a higher risk of compromised physical development and birth defects relative to infants with single-substance exposure. Behavioral outcomes, particularly emotion regulation/reactivity, and physiological outcomes demonstrated a similar pattern. In contrast, other neonatal outcomes (e.g., preterm birth and respiratory distress), and cognition were similar between the prenatal co-exposure and single-substance exposure groups.

**Conclusions:**

This review suggests additive and/or synergistic adverse consequences associated with co-exposure on several outcomes in offspring relative to single substance exposure. These findings highlight the urgent need for prevention and treatment strategies addressing cannabis and NTP use in pregnant women. We discuss the limitations of the included studies and highlight key areas for future research.

## Highlights

• Cannabis and nicotine/tobacco co-use is common during pregnancy.
• Prenatal co-exposure leads to poorer offspring outcomes than single-substance exposure.
• Co-use prevention and treatment strategies are urgently needed for pregnant women.

## 1 Introduction

Changes in cannabis legalization have led to increases in prenatal cannabis use, which may reflect its increased availability and social acceptance (Volkow et al., 2019, Wilkinson et al., 2016). Approximately 11 % of pregnant women in the United States and Canada report past-month non-medical cannabis use, with evidence indicating a rising trend (Kaarid et al., 2020, Volkow et al., 2019, Young-Wolff et al., 2021). The increasing social acceptance of cannabis, along with its perception as a 'natural' or safer alternative to other substances, may be driving its rising use during pregnancy (Chang et al., 2019). However, the absence of established safe levels of prenatal cannabis use (Committee on Obstetric Practice, 2017) raises significant concerns, especially as cannabis potency continues to escalate (ElSohly et al., 2021), potentially amplifying risks to fetal health.

Notably, delta-9-tetrahydrocannabinol (THC), the main psychoactive component in cannabis, readily crosses the placenta and enters the fetal bloodstream (Richardson et al., 2016). Evidence demonstrates that THC interferes with endocannabinoid signaling in the rapidly developing embryonic brain, a critical process for intact neural development (Basavarajappa et al., 2009, Harkany et al., 2007), thereby elevating the risk of adverse fetal outcomes (Cupo et al., 2024). More specifically, prenatal cannabis exposure has been associated with an increased risk of stillbirth, intrauterine growth restriction, and impaired neonatal brain maturation (Lo et al., 2022, Wu et al., 2011). Additionally, data from longitudinal studies suggest that prenatal cannabis exposure may result in long-term behavioral and cognitive consequences (Fried, 2002), which may reflect physiological disturbances in stress and inflammatory responses (Black et al., 2023, Cajachagua-Torres et al., 2021).

Cannabis is commonly co-used with nicotine and tobacco products (NTP). It is estimated that over 70 % of people who use cannabis co-use a NTP (Tucker et al., 2019), with analogous rates reported among pregnant women (Coleman-Cowger et al., 2017). Reasons for NTP co-use include enhancing the euphoric effects of cannabis, minimizing cognitive impairments associated with cannabis use, and attenuating cannabis withdrawal symptoms [see Rabin and George (2015) for review].

Like THC, nicotine crosses the placenta and accumulates in fetal blood, where it disrupts oxygen and nutrient delivery to the fetus (Wickstrom, 2007). Prenatal NTP exposure has been linked to both immediate and long-term adverse outcomes. Short-term consequences include placental abruption, preterm birth, low birth weight, and fetal growth restriction (Ko et al., 2014, Salihu and Wilson, 2007), while long-term effects may involve increased risk of behavioral and cognitive dysfunction (Ernst et al., 2001). These outcomes may be rooted in underlying physiological disturbances, such as dysfunction in the hypothalamic-pituitary adrenal axis (HPA) (He et al., 2017, McDonald et al., 2006, Stroud et al., 2014).

Despite the high prevalence of cannabis and NTP co-use during pregnancy, studies often evaluate their impacts on offspring outcomes separately. This approach fails to test for potential additive or synergistic effects associated with prenatal co-exposure. For instance, both THC and nicotine have been linked to adverse fetal outcomes and disrupted neurodevelopmental trajectories, raising the possibility that their combined use may compound these effects or that one substance may modulate the effects of the other. Given that rates of co-use among pregnant women are rising (Coleman-Cowger et al., 2017), a comprehensive understanding of the interplay between cannabis and NTP on offspring health outcomes is warranted. Therefore, we synthesized findings from both human and animal research on prenatal cannabis and NTP co-exposure, with a focus on neonatal, behavioral, cognitive, and physiological outcomes. We evaluated whether co-exposure was associated with poorer outcomes relative to cannabis-only and NTP-only exposure. Our goal was to fill existing gaps in literature, guide future research, and ultimately inform the development of targeted interventions that more effectively address the complexities of prenatal substance use and its impact on offspring development.

## 2 Methods

### 2.1 Eligibility criteria

Human and animal studies were eligible for inclusion if they (i) assessed prenatal cannabinoid (e.g., THC) exposure and prenatal NTP exposure; and (ii) compared prenatal cannabinoid and NTP co-exposure relative to prenatal cannabinoid-only exposure or prenatal NTP-only exposure on an offspring health-related outcome.

Studies were excluded if they were (i) case studies, reviews, meta-analyses or commentaries; (ii) did not explicitly differentiate between prenatal cannabinoid-only exposure, NTP-only exposure, and co-exposure; (iii) only compared prenatal co-exposure to no prenatal substance exposure.

### 2.2 Search strategies

This protocol was not pre-registered and was conducted following the Preferred Reporting Items for Systematic Reviews and Meta-Analyses (PRISMA) guidelines (Page et al., 2021, Page et al., 2021), see PRISMA checklist in supplementary material. Medline, Embase and PsycINFO databases were searched from inception up to May 12, 2025, using OVID. The search strategy was a combination of keywords such as cannabis/cannabinoids, nicotine/tobacco and pregnancy/prenatal terms. The specific search syntax used for each database can be found in Supplementary Table 1, 2, and 3. Studies were retrieved and screened using the systematic review management platform Covidence. First, duplicates were automatically removed by Covidence. Second, MA and LH independently identified eligible studies based on the titles and abstracts. Third, MA and LH independently screened the selected studies based on full-text reading for final selection. Discrepancies were resolved with discussions with a third author, RAR, to reach consensus.

### 2.3 Quality assessment

The quality of included human studies was assessed using the Joanna Briggs Institute Checklist for Cohort Studies critical appraisal tool and pre-clinical studies were assessed using the Joanna Briggs Institute Checklist for Randomized Control Trials (Moola et al., 2021, Tufanaru et al., 2024). Each component was scored as 1 (yes) or 0 (no or unclear), with total scores calculated by summing individual components. Higher scores indicate greater study quality and lower risk of bias. Quality assessment was conducted independently by two reviewers (LH, MA); discrepancies were resolved through discussion and consensus. The scores from human studies and preclinical studies are presented in Supplementary Table 4 and Supplementary Table 5, respectively.

### 2.4 Data extraction

Data extraction from eligible studies was equally divided between MA and LH. The following study characteristics were extracted from each paper for synthesis: author and year, study design, total sample size and sample size for each exposure group, the range of years in which the children were born, and how cannabinoid and NTP exposure were indexed.

We categorized health-related outcomes into four domains: neonatal outcomes, behavioral outcomes, cognition, and physiological outcomes. For each outcome, we organized the findings as follows: co-exposure compared to single-substance exposure and no-exposure, and single-substance exposure compared to no exposure.

## 3 Results

### 3.1 Study selection

**Fig. 1:**
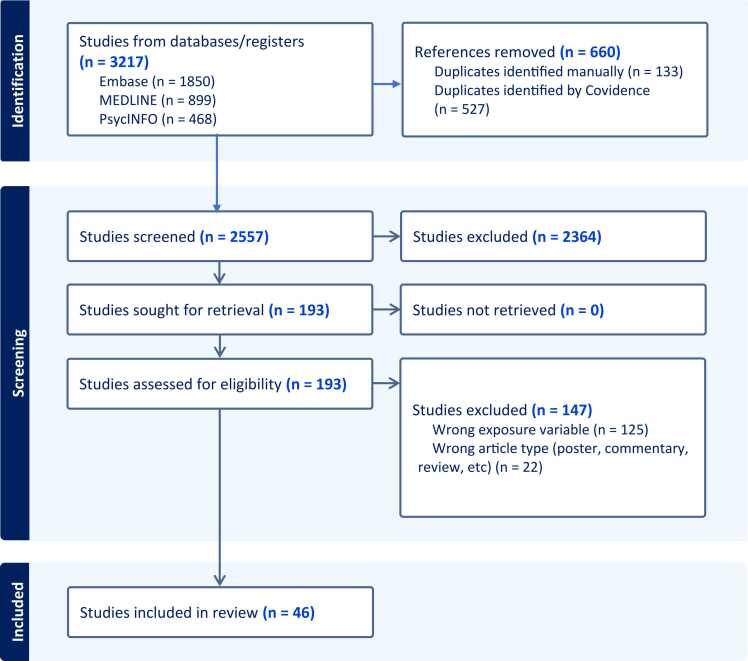
PRISMA flowchart of study selection.

The search identified 3217 potentially relevant records, and 660 records were identified as duplicates. The titles and abstracts of the remaining 2557 records were reviewed. Full-text screening of 193 records led to the exclusion of studies due to wrong article type (n = 22) and wrong exposure variables such as lack of comparison of appropriate substance-using group (n = 125). This resulted in 46 studies eligible for inclusion in the review. The flow diagram of the study selection process is illustrated in Fig. 1.

### 3.2 Study characteristics

Key characteristics of the 46 included studies are summarized in Table 1. Notably, some studies assessed multiple outcomes. This resulted in 21 studies assessing neonatal outcomes, 12 studies assessing behavioral outcomes, 10 studies assessing cognition, and 8 studies assessing physiological outcomes. All studies were rated as good quality, based on the quality assessment conducted (see Supplementary material tables 4,5), indicating a low risk of bias in the evidence synthesized.

**Table 1:** Summary of studies included in review, grouped by primary outcome.

Author, Year	Study Design	Extracted Samples	Birth Years of Children	Assessment used to index cannabis exposure during pregnancy	Assessment used to index NTP exposure during pregnancy
**Neonatal Outcomes**					
Abdelwahab et al. (2022)	Prospective cohort	CO, n = 37 NO, n = 66 CE, n = 74 UE, n = 148	2010–2015	Positive maternal urine toxicology for cannabis	Self-reported use
Bandoli et al. (2021)	Retrospective cohort	CO, n = 15,321 CE, n = 6705 UE, n = 3037,957	2011–2017	Cannabis dependence, non-dependent cannabis use, or cannabis-related disorders (ICD−9-CM 304.3, 305.2; ICD−10-CM F12 diagnostic codes from health record)	Non-dependent tobacco use, tobacco use disorder complicating pregnancy, nicotine dependence (ICD−9-CM 305.1, 649.0; ICD−10-CM O99.33, F17 diagnostic codes from health record)
Breit et al. (2022)	Experimental animal model	NO, n = 12 CO, n = 11 CE, n = 12 UE, n = 13	Not applicable	40-minute daily exposure at 100 mg/ml for gestational days 5–20	40 min daily exposure at 36 mg/ml for gestational days 5–20
Brink et al. (2022)	Prospective cohort	NO, n = 862 CE, n = 64 UE, n = 877	2007–2015	Self-reported use (TLFB)	Self-reported use (TLFB)
Chabarria et al. (2016)	Retrospective cohort	CO, n = 58 NO, n = 194 CE, n = 48 UE, n = 11,769	2011–2015	Self-reported use	Self-reported use
Coleman-Cowger et al. (2018)	Prospective cohort	CO, n = 60 NO, n = 39 CE, n = 45 UE, n = 354	2017–2018	Self-reported use (4 P's plus questionnaire; EMR chart review) or positive maternal urine or hair toxicology for cannabis	Self-reported use (4 P's plus questionnaire; EMR chart review)
Crosland et al. (2024)	Retrospective cohort	CO, n = 23,007 NO, n = 56,811 CE, n = 10,312 UE, n = 3039,129	2012–2019	Cannabis dependence, non-dependent cannabis use, or cannabis-related disorders (ICD−9-CM 304.3, 305.2; ICD−10-CM F12 diagnostic codes from infant birth certificate)	Non-dependent tobacco use, tobacco use disorder complicating pregnancy, nicotine dependence (ICD−9-CM 305.1, 649.0; ICD−10-CM O99.33, F17 diagnostic codes from infant birth certificate)
Dunn et al. (2023)	Retrospective cohort	CO, n = 49 NO, n = 249 CE, n = 32	2019	Self-reported use	Self-reported use
Fried and O'Connell (1987)	Prospective cohort	NR	1981	Self-reported (≥5 joints/week)	Self-reported (>16 mg nicotine/day)
Gray et al. (2010)	Prospective cohort	NO, n = 23 CE, n = 39	2006–2009	Self-reported use or positive maternal oral fluid or positive infant meconium toxicology for cannabis	Self-reported use or positive maternal oral fluid or infant meconium toxicology screen
Haight et al. (2021)	Cross-sectional	HF CO, n = 99 LF CO, n = 78 NO, n = 617 HF CE, n = 165 LF CE, n = 59 UE, n = 5142	2017	Self-reported use (PRAMS); HF: > 1/week; LF: 2–3 times per month or once a month or less	Self-reported use (PRAMS) or indication on infant birth certificate
Hussain et al. (2022)	Experimental animal model	NO, n = 24 CO, n = 22 CE, n = 24 UE, n = 26	Not applicable	40-minute daily exposure at 100 mg/ml for gestational days 5–20	40 min daily exposure at 36 mg/ml for gestational days 5–20
Leemaqz et al. (2016)	Retrospective cohort	CO, n = 315 NO, n = 1473	2044–2011	Self-reported	Self-reported
Nawa et al. (2020)	Retrospective cohort	CO, n = 119 NO, n = 364	1998–2018	Self-reported	Self-reported
Nguyen and Harley (2022)	Retrospective cohort	CO, n = 1228 NO, n = 2644 CE, n = 783 UE, n = 27,720	2017–2019	Self-reported use (PRAMS)	Indication on infant birth certificate
Shi et al. (2021)	Retrospective cohort	CO, n = 15,990 CE, n = 4247 UE, n = 40,474	2001–2012	Cannabis dependence, or non-dependent cannabis use (ICD−9-CM 304.3, 305.20 diagnostic codes from delivery discharge record)	Self-reported use (infant birth certificate)
Schuetze et al. (2018)	Prospective cohort	NR	2006–2008	Self-reported use (TLFB) or positive maternal oral fluid or infant meconium toxicology screen	Self-reported use (TLFB) or positive maternal oral fluid or infant meconium toxicology screen
Sturrock et al. (2020)	Retrospective cohort	NR	2017–2018	Self-reported use (medical record)	Self-reported use (medical record)
Waddell et al. (2024)	Retrospective cohort	NO, n = 71 CE, n = 127	2016–2021	Self-reported use or positive maternal/infant urine toxicology for cannabis or cord blood analysis	Self-reported use
Warshak et al. (2015)	Retrospective cohort	CO, n = 153 NO, n = 1214 CE, n = 208 UE, n = 6107	2008–2011	Self-reported use or positive toxicology screen (unspecified) for cannabis (medical record)	Self-reported use (medical record)
**Behavioral Outcomes**					
Eiden et al. (2018)	Prospective cohort	NO, n = 81 CE, n = 97 UE, n = 69	NR	Self-reported use (TLFB) or positive maternal oral fluid or infant meconium toxicology for cannabis	Self-reported use (TLFB) or positive maternal oral fluid or infant meconium toxicology for cannabis
Eiden et al. (2018)	Prospective cohort	NO, n = 75 CE, n = 103 UE, n = 69	NR	Self-reported use (TLFB) or positive maternal oral fluid or infant meconium toxicology screen	Self-reported use (TLFB) or positive maternal oral fluid or infant meconium toxicology screen
Garrison-Desany et al. (2022)	Prospective cohort	NR	1998–2019	Self-reported use	Self-reported use
Godleski et al. (2016)	Prospective cohort	NR	NR	Self-reported use (TLFB) or positive maternal oral fluid or infant meconium toxicology screen	Self-reported use (TLFB) or positive maternal oral fluid or infant meconium toxicology screen
Godleski et al. (2018)	Prospective cohort	NO, n = 81 CE, n = 97 UE, n = 69	NR	Self-reported use (TLFB) or positive maternal oral fluid or infant meconium toxicology screen	Self-reported use (TLFB) or positive maternal oral fluid or infant meconium toxicology screen
Kelm et al. (2024)	Prospective cohort	NO, n = 75 CE, n = 103 UE, n = 69	NR	Self-reported use (TLBF) and positive maternal oral fluid	Self-reported use (TLBF) and positive maternal oral fluid
Lallai et al. (2022)	Experimental animal model	NR	Not applicable	5 mg/kg THC orally daily, five days before mating until day 20 of gestation	5 mg/ml nicotine e-cigarette vapour across 1 h with 5-sec vapour puffs at 5 min intervals to achieve > 22 ng/ml blood cotinine levels
Nadler et al. (2025)	Prospective cohort	CO, n = 225 NO, n = 966 CE, n = 290 UE, n = 8311	NR	Self-reported use	Self-reported use
Nutor et al. (2024)	Prospective cohort	CO, n = 187 CE, n = 162 UE, n = 5066	1997–2020	Self-reported use and positive toxicology screen	Self-reported use
Perry et al. (2024)	Prospective cohort	NO, n = 81 CE, n = 97 UE, n = 69	NR	Self-reported use (TLFB) or positive maternal oral fluid or infant meconium toxicology screen	Self-reported use (TLFB) or positive maternal oral fluid or infant meconium toxicology screen
Schuetze et al. (2018)	Prospective cohort	NR	2006–2008	Self-reported use (TLFB) or positive maternal oral fluid or infant meconium toxicology screen	Self-reported use (TLFB) or positive maternal oral fluid or infant meconium toxicology screen
Stroud et al. (2018)	Prospective cohort	NO, n = 45 CE, n = 24 UE, n = 42	2006–2010	Self-reported use (TLFB) or expired alveolar CO or infant meconium with ≥ 10 ng/g cannabinoid markers	Self-reported use (TLFB) or expired alveolar CO or infant meconium with ≥ 10 ng/g nicotine markers or presence of cotinine in maternal oral fluid
**Cognitive Outcomes**					
Fried et al. (1992)	Prospective cohort	NR	1980–1983	Self-reported use of > 6 joints/week	Self-reported use of > 15 mg nicotine/day
Fried and Watkinson, (1988)	Prospective cohort	NR	1980–1983	Self-reported use	Self-reported use of > 15 mg nicotine/day
Fried and Watkinson, (1990)	Prospective cohort	NR	1980–1983	Self-reported use of > 6 joints/week	Self-reported use of > 15 mg nicotine/day
Fried and Watkinson, (2000)	Prospective cohort	NR	1978-NR	Self-reported use of > 6 joints/week	Self-reported use
Fried et al. (1992)	Prospective cohort	NR	1980–1983	Self-reported use of > 6 joints/week	Self-reported use of > 15 mg nicotine/day
Lallai et al. (2022)	Experimental animal model	NR	Not applicable	5 mg/kg THC orally daily, five days before mating until day 20 of gestation	5 mg/ml nicotine e-cigarette vapour across 1 h with 5-sec vapour puffs at 5 min intervals to achieve > 22 ng/ml blood cotinine levels
Richardson et al. (1995)	Prospective cohort	NR	1983–1986	Self-reported use	Self-reported use
Shisler et al. (2024)	Prospective cohort	NO, n = 37 CE, n = 62 UE, n = 34	NR	Self-reported use (TLFB) or positive maternal oral fluid or infant meconium toxicology screen	Self-reported use (TLFB) or positive maternal oral fluid or infant meconium toxicology screen
Stroud et al. (2018)	Prospective cohort	NO, n = 45 CE, n = 24 UE, n = 42	2006–2010	Self-reported use (TLFB) or expired alveolar CO or infant meconium with ≥ 10 ng/g cannabinoid markers	Self-reported use (TLFB) or expired alveolar CO or infant meconium with ≥ 10 ng/g nicotine markers or presence of cotinine in maternal oral fluid
Willford et al. (2010)	Prospective cohort	NR	NR	Self-reported use	Self-reported use
**Physiological Outcomes**					
Eiden et al. (2020)	Prospective/retrospective cohort	NO, n = 67 CE, n = 83 UE, n = 88	2007-NR	Self-reported use (TLFB) or positive maternal oral fluid or infant meconium toxicology screen	Self-reported use (TLFB) or positive maternal oral fluid or infant meconium toxicology screen
Eiden et al. (2018)	Prospective cohort	NO, n = 81 CE, n = 97 UE, n = 69	NR	Self-reported use (TLFB) or positive maternal oral fluid or infant meconium toxicology for cannabis	Self-reported use (TLFB) or positive maternal oral fluid or infant meconium toxicology for cannabis
Kong et al. (2023)	Prospective cohort	NO, n = 83 CE, n = 107 UE, n = 69	2007–2008	Self-reported use (TLFB) or positive maternal oral fluid or infant meconium toxicology screen	Self-reported use (TLFB) or positive maternal oral fluid or infant meconium toxicology screen
Molnar et al. (2018)	Prospective cohort	NO, n = 16 CE, n = 17 UE, n = 12	NR	Self-reported use (TLFB) or positive maternal oral fluid or infant meconium toxicology screen	Self-reported use (TLFB) or positive maternal oral fluid or infant meconium toxicology screen
Schuetze et al. (2019)	Prospective cohort	NO, n = 81 CE, n = 97 UE, n = 69	NR	Self-reported use (TLFB) or positive maternal oral fluid or infant meconium toxicology screen	Self-reported use (TLFB) or positive maternal oral fluid or infant meconium toxicology screen
Simon et al. (2023)	Prospective/retrospective cohort	NO, n = 64 CE, n = 68 UE, n = 79	2007-NR	Self-reported use (TLFB) or positive maternal oral fluid or infant meconium toxicology screen	Self-reported use (TLFB) or positive maternal oral fluid or infant meconium toxicology screen
Stroud et al. (2018)	Prospective cohort	NO, n = 45 CE, n = 24 UE, n = 42	2006–2010	Self-reported use (TLFB) or expired alveolar CO or infant meconium with ≥ 10 ng/g cannabinoid markers	Self-reported use (TLFB) or expired alveolar CO or infant meconium with ≥ 10 ng/g nicotine markers or presence of cotinine in maternal oral fluid
Stroud et al. (2020)	Prospective cohort	NO, n = 45 CE, n = 24 UE, n = 42	2006–2010	Self-reported use (TLFB) or positive maternal oral fluid or infant meconium toxicology screen	Self-reported use (TLFB) or positive maternal oral fluid or infant meconium toxicology screen

**Acronyms** NTP: Nicotine/Tobacco products; CO: Cannabis only exposure; NO: NTP only exposure; CE: Co-exposure; UE: Unexposed control; ICD-9-CM: International Classification of Diseases, 9th Revision, Clinical Modification; ICD-10-CM: International Classification of Diseases, 10th Revision, Clinical Modification; TLFB: Timeline Follow Back Interview; EMR: Electronic medical records; NR: Not reported; HF: High frequency; LF: Low frequency; PRAMS: Pregnancy Risk Assessment Monitoring System.

### 3.3 Neonatal outcomes

A total of 21 studies examined neonatal outcomes [humans studies (n = 19) and preclinical studies (n = 2)], which included (very) preterm birth, physical development [including measures of small for gestational age (SGA), fetal growth restriction (FGR), birth weight, birth length and head circumference, eye opening and motor development], admission to the neonatal intensive care unit (NICU), presence of birth defects, and newborn health measured via the APGAR (appearance, pulse, grimace, activity, and respiration) score and respiratory distress syndrome (Abdelwahab et al., 2022, Bandoli et al., 2021, Breit et al., 2022, Brink et al., 2022, Chabarria et al., 2016, Coleman-Cowger et al., 2018, Crosland et al., 2024, Dunn et al., 2023, Fried and O′Connell, 1987, Gray et al., 2010, Haight et al., 2021, Hussain et al., 2022, Leemaqz et al., 2016, Massey et al., 2018, Nawa et al., 2020, Nguyen and Harley, 2022, Schuetze et al., 2018, Shi et al., 2021, Sturrock et al., 2020, Waddell et al., 2024, Warshak et al., 2015).

In human studies, substance use was predominantly evaluated using self-report assessments [(n = 13) (Abdelwahab et al., 2022, Brink et al., 2022, Chabarria et al., 2016, Coleman-Cowger et al., 2018, Dunn et al., 2023, Fried and O′Connell, 1987, Haight et al., 2021, Leemaqz et al., 2016, Nawa et al., 2020, Nguyen and Harley, 2022, Sturrock et al., 2020, Waddell et al., 2024, Warshak et al., 2015)]. Five studies combined maternal self-report with biological verification (Coleman-Cowger et al., 2018, Gray et al., 2010, Massey et al., 2018, Schuetze et al., 2018, Warshak et al., 2015), while three studies relied on birth certificates, maternal health records and/or hospital discharge papers to ascertain maternal substance use (Bandoli et al., 2021, Crosland et al., 2024, Shi et al., 2021).

#### 3.3.1 Preterm and very preterm birth

Twelve studies examined the association between prenatal co-exposure and preterm birth (Bandoli et al., 2021, Brink et al., 2022, Chabarria et al., 2016, Coleman-Cowger et al., 2018, Crosland et al., 2024, Dunn et al., 2023, Haight et al., 2021, Leemaqz et al., 2016, Nawa et al., 2020, Nguyen and Harley, 2022, Shi et al., 2021, Warshak et al., 2015), defined as birth before 37 weeks of gestational age. Other studies reported on very preterm birth, defined as birth before 32 weeks of gestational age (Bandoli et al., 2021, Brink et al., 2022, Chabarria et al., 2016, Crosland et al., 2024).

Prenatal co-exposure was associated with greater risk of preterm and very preterm birth compared to prenatal cannabis-only exposure (Bandoli et al., 2021, Crosland et al., 2024, Dunn et al., 2023, Nawa et al., 2020, Shi et al., 2021), prenatal tobacco-only exposure (Crosland et al., 2024, Dunn et al., 2023, Nawa et al., 2020), or no prenatal substance exposure (Bandoli et al., 2021, Chabarria et al., 2016, Crosland et al., 2024, Dunn et al., 2023, Nawa et al., 2020, Nguyen and Harley, 2022). In contrast, other studies found no association between prenatal co-exposure and preterm birth (Brink et al., 2022, Coleman-Cowger et al., 2018, Haight et al., 2021, Leemaqz et al., 2016, Warshak et al., 2015), or very preterm birth (Chabarria et al., 2016) relative to all other exposure groups.

Compared to unexposed infants, there was some evidence that infants with cannabis-only exposure had increased risk for preterm birth (Bandoli et al., 2021, Crosland et al., 2024, Leemaqz et al., 2016, Nawa et al., 2020, Shi et al., 2021), however, the majority of studies reported no association (Brink et al., 2022, Chabarria et al., 2016, Coleman-Cowger et al., 2018, Dunn et al., 2023, Haight et al., 2021, Nguyen and Harley, 2022, Warshak et al., 2015). In contrast, there was more consistent evidence linking prenatal tobacco-only exposure to increased risk for preterm birth (Brink et al., 2022, Chabarria et al., 2016, Crosland et al., 2024, Dunn et al., 2023, Leemaqz et al., 2016, Nawa et al., 2020, Nguyen and Harley, 2022, Shi et al., 2021) as only one study reported no association (Coleman-Cowger et al., 2018). Notably, this comparison was not conducted in all studies (Bandoli et al., 2021, Haight et al., 2021, Warshak et al., 2015).

#### 3.3.2 Physical development

Nineteen studies examined the association between co-exposure and infant physical development. Physical development was assessed using SGA and FGR, referring to infants weight or growth measurements under the 10th percentile for their gestational age, respectively; and markers of fetal growth such as birth weight, length, and head circumference (Abdelwahab et al., 2022, Bandoli et al., 2021, Brink et al., 2022, Chabarria et al., 2016, Coleman-Cowger et al., 2018, Crosland et al., 2024, Dunn et al., 2023, Fried and O′Connell, 1987, Gray et al., 2010, Haight et al., 2021, Massey et al., 2018, Nguyen and Harley, 2022, Schuetze et al., 2018, Shi et al., 2021, Sturrock et al., 2020, Waddell et al., 2024, Warshak et al., 2015). Two animal studies investigated the effects of nicotine and/or THC administration on offspring birth weight, eye opening (Breit et al., 2022), and motor development (Hussain et al., 2022).

Five studies reported that prenatal co-exposure increased the risk for SGA or FGR relative to cannabis-only (Abdelwahab et al., 2022, Bandoli et al., 2021, Crosland et al., 2024, Dunn et al., 2023) or tobacco-only exposure (Abdelwahab et al., 2022, Crosland et al., 2024, Dunn et al., 2023, Nguyen and Harley, 2022). Contrasting this, other studies found no difference in SGA or FGR risk between co-exposure and cannabis-only exposure (Chabarria et al., 2016, Haight et al., 2021, Shi et al., 2021, Warshak et al., 2015) or co-exposure and tobacco-only exposure groups (Chabarria et al., 2016, Haight et al., 2021, Waddell et al., 2024, Warshak et al., 2015), though not all studies included both comparison groups (Shi et al., 2021, Waddell et al., 2024).

Compared to unexposed infants, prenatal co-exposure was linked to higher SGA or FGR risk in most studies (Abdelwahab et al., 2022, Bandoli et al., 2021, Crosland et al., 2024, Nguyen and Harley, 2022, Shi et al., 2021, Warshak et al., 2015), though two studies found no association (Brink et al., 2022, Haight et al., 2021).

Most studies reported greater SGA or FGR risk for prenatal cannabis-only exposure relative to unexposed infants (Abdelwahab et al., 2022, Bandoli et al., 2021, Dunn et al., 2023, Haight et al., 2021, Shi et al., 2021, Warshak et al., 2015), though other studies reported no association for SGA (Chabarria et al., 2016, Dunn et al., 2023, Haight et al., 2021, Nguyen and Harley, 2022) or FGR risk (Warshak et al., 2015). Similarly, prenatal tobacco-only exposure relative to no exposure elevated SGA (Crosland et al., 2024, Nguyen and Harley, 2022) or FGR risks (Brink et al., 2022, Dunn et al., 2023), while two studies reported null findings (Abdelwahab et al., 2022, Chabarria et al., 2016). Notably, some studies did not include a cannabis-only (Brink et al., 2022, Waddell et al., 2024) or a tobacco-only comparison group (Bandoli et al., 2021, Shi et al., 2021, Warshak et al., 2015).

Nine studies reported that prenatal co-exposure was associated with an increased risk of low birth weight (Brink et al., 2022, Chabarria et al., 2016, Dunn et al., 2023, Gray et al., 2010, Haight et al., 2021, Nguyen and Harley, 2022, Schuetze et al., 2018, Shi et al., 2021, Sturrock et al., 2020) and head circumference (Chabarria et al., 2016, Gray et al., 2010, Schuetze et al., 2018, Sturrock et al., 2020) relative to exposure to either substance alone. Consistent with these findings, co-exposed infants also had higher risk of low birth length (Schuetze et al., 2018), shorter length at 1 year old (Brink et al., 2022), and reduced head circumference (Brink et al., 2022, Chabarria et al., 2016, Coleman-Cowger et al., 2018, Schuetze et al., 2018) compared to unexposed infants. Notably, Haight et al. (2021) found that this association was only significant when mothers used cannabis at a high frequency (at least weekly) relative to low frequency (< 3 times/month). Interestingly, one study reported that co-exposed infants had higher birth weight compared to infants with tobacco-only exposure and unexposed infants (Fried and O'Connell, 1987). Further, four studies reported that co-exposed infants were at similar risk for abnormal fetal growth markers (birth length, birth weight, and head circumference) relative to tobacco-only exposure (Massey et al., 2018, Waddell et al., 2024) or unexposed infants (Brink et al., 2022, Coleman-Cowger et al., 2018, Massey et al., 2018).

Prenatal cannabis-only exposure compared to unexposed infants also amplified the risk of low birth weight (Dunn et al., 2023, Gray et al., 2010, Haight et al., 2021, Massey et al., 2018, Nguyen and Harley, 2022, Shi et al., 2021), birth length and head circumference (Dunn et al., 2023, Gray et al., 2010); though two studies observed null effects (Coleman-Cowger et al., 2018, Sturrock et al., 2020). Prenatal tobacco-only exposure was also associated with an increased risk of low birth weight and reductions in other fetal growth makers (i.e., birth length, head circumference) relative to unexposed infants (Brink et al., 2022, Chabarria et al., 2016, Dunn et al., 2023, Massey et al., 2018, Nguyen and Harley, 2022, Schuetze et al., 2018, Sturrock et al., 2020); though one study reported no association (Coleman-Cowger et al., 2018).

Finally, two recent preclinical studies compared the effects of prenatal nicotine exposure (36 mg/ml), THC exposure (100 mg/ml), combined nicotine and THC, and vehicle exposure on neonatal developmental outcomes. Birth weight and eye opening were assessed in the first study, and no significant effects emerged across all exposure groups (Breit et al., 2022). In the second study, co-exposure was associated with greater delay in sensorimotor development compared to either substance alone and vehicle exposure. Furthermore, prenatal exposure to either THC or nicotine impaired motor coordination, while combined exposure exacerbated these effects compared to either substance alone and vehicle, particularly among females (Hussain et al., 2022).

#### 3.3.3 NICU admission

Seven studies examined the impact of prenatal co-exposure on admissions to the NICU (Bandoli et al., 2021, Coleman-Cowger et al., 2018, Crosland et al., 2024, Shi et al., 2021, Waddell et al., 2024, Warshak et al., 2015) or the special care nursery (Dunn et al., 2023). Two studies revealed that infants with prenatal co-exposure had more frequent NICU admissions compared to cannabis-only exposure (Bandoli et al., 2021, Crosland et al., 2024), tobacco-only exposure (Crosland et al., 2024), or no exposure (Bandoli et al., 2021, Crosland et al., 2024). However, several other studies reported comparable risk between prenatally co-exposed infants and infants with cannabis-only exposure (Shi et al., 2021, Warshak et al., 2015), tobacco-only exposure (Waddell et al., 2024, Warshak et al., 2015), or no exposure (Coleman-Cowger et al., 2018, Dunn et al., 2023).

Results comparing either substance alone to no substance exposure were similarly mixed. Three studies noted NICU admissions were more frequent in infants with prenatal cannabis-only (Crosland et al., 2024, Warshak et al., 2015), or tobacco-only exposure (Dunn et al., 2023) relative to unexposed infants, while other studies reported similar risk for cannabis-only (Coleman-Cowger et al., 2018, Dunn et al., 2023, Shi et al., 2021) or tobacco-only exposure (Coleman-Cowger et al., 2018).

#### 3.3.4 Presence of birth defects

Three studies examined the association between prenatal co-exposure and major birth defects (Bandoli et al., 2021, Coleman-Cowger et al., 2018, Warshak et al., 2015).

Birth malformations, such as cardiac, musculoskeletal, or gastrointestinal defects, were investigated by Coleman-Cowger et al. (2018). Investigators reported that the risk of birth defects were three times greater in infants with prenatal co-exposure relative to unexposed infants, whereas either substance exposure alone did not increase the risk of birth defects (Coleman-Cowger et al., 2018).

Bandoli et al. (2021) observed a 53 % increased risk of central nervous system and gastrointestinal malformations in infants with prenatal co-exposure relative to cannabis-only exposure. A 46 % increased risk of these malformations were observed in infants with prenatal exposure to cannabis-only exposure relative to unexposed infants. Notably, this study did not include a tobacco-only exposure group (Bandoli et al., 2021).

Warshak et al. (2015) explored birth anomalies, defined as a significant anomaly noted on the delivery admission history that required neonatal evaluation or intervention, in infants with prenatal co-exposure, cannabis-only exposure, tobacco-only exposure, and unexposed infants. No group differences were found across groups (Warshak et al., 2015).

#### 3.3.5 Newborn health: APGAR scores and respiratory distress

APGAR is a standardized test used to assess a newborn’s health shortly after birth by measuring factors such as heart rate, respiration, and muscle tone. All five studies included found no significant difference in APGAR scores between prenatal co-exposure, cannabis-only, tobacco-only, and unexposed groups (Chabarria et al., 2016, Coleman-Cowger et al., 2018, Dunn et al., 2023, Gray et al., 2010, Waddell et al., 2024).

Notably, a later study investigated the relationship between prenatal co-exposure and respiratory distress syndrome, which occurs when an infant’s lungs are not fully developed causing trouble breathing. Investigators found that infants with prenatal co-exposure were at the highest risk for respiratory distress syndrome compared to exposure to either substance alone or no-exposure (Crosland et al., 2024). Contrasting these results, a study by Waddell and colleagues found no group differences in respiratory distress between co-exposed infants and infants exposed to tobacco alone. This study lacked a cannabis-only and unexposed groups (Waddell et al., 2024).

### 3.4 Behavioral outcomes

The association between prenatal co-exposure on childhood behavioral outcomes relative to prenatal cannabis and NTP exposure was explored in 12 studies: 11 human (Eiden et al., 2018, Eiden et al., 2018, Garrison-Desany et al., 2022, Godleski et al., 2016, Godleski et al., 2018, Kelm et al., 2024, Nadler et al., 2025, Nutor et al., 2024, Perry et al., 2024, Schuetze et al., 2018, Stroud et al., 2018) and one preclinical study (Lallai et al., 2022). We discussed these studies in terms of internalizing and externalizing behaviors and emotion regulation/reactivity. However, one study examined autism spectrum disorder diagnoses and traits, which we described separately (Nutor et al., 2024).

Two human studies relied on self-report for determining prenatal substance use (Garrison-Desany et al., 2022, Nadler et al., 2025) and nine studies combined maternal self-report with biological verification (Eiden et al., 2018, Eiden et al., 2018, Godleski et al., 2016, Godleski et al., 2018, Kelm et al., 2024, Nutor et al., 2024, Perry et al., 2024, Schuetze et al., 2018, Stroud et al., 2018).

#### 3.4.1 Internalizing behaviors

Using the Childhood Behavior Checklist (CBCL) to index internalizing symptoms, Eiden et al. (2018) observed higher levels of anxiety, depression, and social withdrawal in female toddlers with prenatal co-exposure compared to female toddlers with tobacco-only exposure and unexposed toddlers (Eiden et al., 2018); these associations were not present in three-year old boys. A cannabis-only exposure group was not included. Contrasting this, a subsequent preclinical study noted opposite sex differences in anxiety in rats during the adolescence (Lallai et al., 2022). Males with prenatal co-exposure had greater anxiety relative to unexposed males but did not differ from males with single substance exposure. No differences in anxiety-associated behavior were observed in female adolescent rats.

Other studies reported no group differences between co-exposure, single substance exposure, and unexposed in toddlers using the Brief Infant Toddler Social Emotional Assessment scale in toddlers (Godleski et al., 2016) or in children using the CBCL (Nadler et al., 2025). However, Nadler et al. (2025) found an interaction between tobacco consumption and cannabis consumption on internalizing behaviors such that greater tobacco use amplified the positive association between the amount of prenatal cannabis exposure and severity of internalizing symptoms.

#### 3.4.2 Externalizing behaviors

In a large cohort of children, those with prenatal co-exposure had greater CBCL-derived externalizing symptoms compared to children with prenatal cannabis-only exposure, tobacco-only exposure, and unexposed children (Nadler et al., 2025). Children with cannabis-only and tobacco-only exposure also had greater externalizing scores than unexposed children, but these scores did not differ from each other. Like internalizing symptoms, greater prenatal tobacco exposure strengthened the association between amount of cannabis exposure and externalizing behaviors (Nadler et al., 2025). These findings are at odds with an earlier study that found no association between co-exposure or single substance exposure and externalizing scores using the Brief Infant Toddler Social Emotional Assessment scale in children between 24 and 36 months (Godleski et al., 2018). Another study (median age = 12) that specifically examined risk of childhood ADHD reported that prenatal tobacco exposure was associated with a higher risk of developing the disorder, whereas cannabis-only exposure or co-exposure did not show the same effect (Garrison-Desany et al., 2022).

#### 3.4.3 Emotion regulation/reactivity

Stroud et al. (2018) found that infants with prenatal co-exposure had poorer self-regulation and increased need for external soothing as indexed by the NICU Network Neurobehavioral Scale compared to tobacco exposed infants and unexposed infants (Stroud et al., 2018). A cannabis-only exposure group was not included.

Three studies using similar cohorts found no group differences between toddlers with prenatal co-exposure, tobacco-only exposure and no exposure on measures of emotion regulation and emotional reactivity (Eiden et al., 2018, Perry et al., 2024, Schuetze et al., 2018). Further, prenatal substance exposure did not directly predict emotion regulation in these samples (Eiden et al., 2018, Perry et al., 2024, Schuetze et al., 2018). However, another study found that among male toddlers, those with prenatal co-exposure exhibited blunted reactivity compared to those exposed to tobacco alone or not exposed at all, a pattern not observed among female toddlers (Kelm et al., 2024). Notably, cannabis-only exposure groups were not included in these studies.

#### 3.4.4 Autism spectrum traits

In a large sample of children and adolescents (N = 11,570) aged 1–18, prenatal tobacco exposure was associated with greater autism-related behaviors as measured by the CBCL and the Social Responsiveness Scale as well as increased likelihood of child autism spectrum disorder (Nutor et al., 2024). However, interactions between prenatal cannabis and tobacco exposure did not show any significant effects on these outcomes.

### 3.5 Cognitive outcomes

Nine human (Fried et al., 1992, Fried and Watkinson, 1988, Fried and Watkinson, 1990, Fried and Watkinson, 2000, Fried et al., 1992, Richardson et al., 1995, Shisler et al., 2024, Stroud et al., 2018, Willford et al., 2010) and one preclinical (Lallai et al., 2022) study examined cognition in offspring with prenatal co-exposure relative to single substance exposure. All human studies assessed prenatal substance use via self-report, except one which also included biological assay of maternal saliva and infant meconium (Shisler et al., 2024).

Five studies leveraged data from the Ottawa Prenatal Prospective Study, a cohort of low-risk women recruited between 1979 and 1983 from prenatal clinics across Ottawa, Ontario (Canada). These studies were primarily designed to explore the effects of prenatal cannabis exposure in children between the age of 12-months and 12 years old (Fried et al., 1992, Fried and Watkinson, 1988, Fried and Watkinson, 1990, Fried and Watkinson, 2000, Fried et al., 1992). A similar cohort was analyzed by Richardson et al. (1995) to assess developmental outcomes at 9 and 19 months. Willford et al. (2010) used data from the Maternal Health Practices and Child Development project, a study of at-risk women recruited early in pregnancy from a prenatal clinic in Pittsburgh, PA, USA. Stroud and colleagues (2018) investigated infant neurobehavioral development during the first postnatal month in a diverse, low-income sample. Shisler et al. (2024) examined attention and working memory at multiple time points in children from 2 months to kindergarten age. Lastly, a preclinical study by Lallai et al. (2022) examined the effects of prenatal co-exposure throughout pregnancy in rats, focusing on outcomes during adolescence.

The majority of these studies found no interaction between prenatal cannabis exposure and prenatal tobacco exposure on cognition using an array of batteries (e.g., McCarthy Scales of Children’s Abilities, Bayley Scales of Infant Development, Test of Visual-Perceptual Skills) (Fried et al., 1992, Fried and Watkinson, 1988, Fried and Watkinson, 1990, Fried and Watkinson, 2000, Fried et al., 1992, Richardson et al., 1995, Willford et al., 2010). While all studies reported negative effects of prenatal tobacco exposure on cognition, one study did not find negative effects of prenatal cannabis exposure on cognition in 5- and 6-year old children (Fried et al., 1992). Unexpectedly, in 12-month old children, prenatal cannabis exposure was positively associated with attention span, goal-directedness, object orientation, reactivity, and vocalization (Fried and Watkinson, 1988).

A later study examined attention in infants within the first month after birth and found that infants with prenatal co-exposure and tobacco-only exposure had poorer attention compared to unexposed newborns (Stroud et al., 2018); a cannabis-only exposure group was not included.

While no significant difference emerged between co-exposure and tobacco-only exposure, notably the impact of co-exposure on attentional dysfunction was ~40 % greater than the impact of tobacco alone. Sex effects were examined, but no significant group differences emerged. Similarly, another study investigated sustained attention, attentional shift, and working memory in kindergarten-aged children. There were no difference between co-exposed children and children exposed to tobacco-only on attentional or working memory tasks. Unexpectedly, the authors reported that compared to unexposed children, those with prenatal co-exposure had higher scores on a short-term memory task. A cannabis-only exposure group was not included (Shisler et al., 2024).

A subsequent preclinical study also examined sex effects of prenatal THC and/or nicotine exposure throughout pregnancy on short-term memory and sensorimotor gating using prepulse inhibition (PPI) in rats during adolescence (Lallai et al., 2022). Deficits in short-term memory were found in males prenatally exposed to THC, either alone or with nicotine, and in females exposed to THC alone. Among adolescent males, co-exposure elicited a deficit in PPI that was not observed in the presence of either nicotine or THC alone. For females, prenatal nicotine exposure treatment resulted in enhanced PPI compared to the unexposed group (Lallai et al., 2022).

### 3.6 Physiological outcomes

Eight studies examined the association between prenatal co-exposure and physiological outcomes in offspring which included cortisol (Eiden et al., 2020, Stroud et al., 2020), immune and inflammatory biomarkers (Molnar et al., 2018, Simon et al., 2023), autonomic regulation (Eiden et al., 2018, Schuetze et al., 2019), motor activity, (Stroud et al., 2018) and anthropometrics (Kong et al., 2023, Simon et al., 2023). All eight studies combined maternal self-report with biological verification.

#### 3.6.1 Cortisol

Two studies examined cortisol and cortisol reactivity in infants and children with co-exposure, tobacco-only exposure, and no exposure (Eiden et al., 2020, Stroud et al., 2020). During the first postnatal month, Stroud et al. (2020) observed that infant males, but not females, with prenatal co-exposure had lower cortisol values at rest relative to male infants with tobacco-only exposure and unexposed male infants (Stroud et al., 2020). Similarly, another study in kindergarten-aged children showed that, relative to unexposed children, those with prenatal co-exposure exhibited a flattened cortisol response following exposure to a laboratory stressor (Eiden et al., 2020). No difference was found between the tobacco-only exposure group and the unexposed group.

#### 3.6.2 Immune and inflammatory biomarkers

Molnar et al. (2018) compared salivary Secretory Immunoglobulin A (SIA), an antibody implicated in immune functioning of mucus membranes by preventing pathogens from adhering and entering epithelial mucosa (Mantis et al., 2011) in kindergarten-aged children with prenatal co-exposure, tobacco-only exposure, and no substance exposure (Molnar et al., 2018). Findings demonstrated that children with co-exposure and tobacco-only exposure had higher SIA levels compared to the non-exposed children. No dose-response associations were found with substance exposure and SIA.

A study from the same group investigated salivary C-reactive protein concentrations, a marker of inflammation, in the same cohort of children (Simon et al., 2023). The authors found a significant interaction between amount of tobacco exposure and amount of cannabis exposure during the third trimester on salivary C-reactive protein concentrations in kindergarten-aged children. At high tobacco exposure, the effect of cannabis exposure was negligible, whereas at low tobacco exposure, there was a positive association between cannabis exposure and C-reactive protein concentrations (Simon et al., 2023).

#### 3.6.3 Autonomic regulation

A study by Eiden et al. (2018) indexed autonomic regulation in 9-month-old infants with prenatal co-exposure, tobacco-only exposure, and no substance exposure using respiratory sinus arrhythmia, a measure of nervous system activity and a reliable psychophysiological index of emotion regulation (Porges, 2007). The study found that co-exposure was associated with a maladaptive increase in respiratory sinus arrhythmia in response to stress during infancy compared to unexposed infants (Eiden et al., 2018). The tobacco-only exposure group did not differ from the co-exposed or the unexposed group. The same group of investigators examined the relationships between prenatal co-exposure, tobacco-only exposure, and no substance exposure using respiratory sinus arrhythmia in 16-month-old infants. The analyses revealed no direct associations between prenatal exposure and regulatory processes (Schuetze et al., 2019). No cannabis-only exposure groups were included in these studies.

#### 3.6.4 Motor activity

Stroud et al. (2018) investigated the association between prenatal co-exposure and infant lethargy during the first postnatal month (Stroud et al., 2018). Lethargy is a measure of low levels of motor, state, and physiologic reactivity as assessed by the NICU Network Neurobehavioral Scale (Lester et al., 2004). Results showed that co-exposed infants had increased lethargy compared to unexposed infants; no significant difference was observed between co-exposed infants and tobacco-only exposed infants. A cannabis-only exposed group was not included.

#### 3.6.5 Anthropometrics

Two studies investigated group differences between children with prenatal co-exposure, tobacco-only exposure, and no exposure on BMI (Body Mass Index)-related outcomes (Kong et al., 2023, Simon et al., 2023). Simon et al. (2023) found no group differences in BMI in kindergarten-aged children, although no covariates were employed to control for potentially confounding variables including sex of the child and mother’s BMI (Simon et al., 2023). In a longitudinal study, Kong et al. (2023) investigated BMI trajectories from birth to middle-childhood (9–12 years) using data from a New York–based prospective cohort (Kong et al., 2023). Findings indicated that co-exposure and tobacco-only exposure were associated with a greater increase in BMI from birth to middle childhood compared to unexposed children. By middle childhood, co-exposed children had higher odds of obesity and greater fat mass than unexposed children; differences were not observed between co-exposed and tobacco-only exposed children. While dose-dependent relationships between prenatal substance exposure and BMI trajectory were investigated, no significant associations emerged.

## 4 Discussion

### 4.1 Summary of principal findings

To our knowledge, this is the first comprehensive review directly comparing the effects of prenatal cannabis and NTP co-exposure with those of cannabis-only and NTP-only exposure on neonatal, behavioral, cognitive, and physiological outcomes in offspring.

Converging evidence indicates that co-exposure is associated with select adverse neonatal outcomes that are more pronounced that those observed with cannabis-only and NTP-only exposure. Infants with prenatal co-exposure had a higher risk of impaired physical development compared to those exposed to either substance alone, as indicated by fetal growth markers (e.g., birth weight, length, head circumference) (Chabarria et al., 2016, Dunn et al., 2023, Gray et al., 2010, Schuetze et al., 2018, Shi et al., 2021, Sturrock et al., 2020); evidence was less consistent when assessed using SGA and FGR, given that 50 % of studies reported null findings. This suggests that fetal growth markers may be more sensitive in detecting exposure-related impairments than categorical measures with strict cutoffs. We also found that co-exposed neonates had a greater incidence of birth malformations relative to single-substance exposure (Bandoli et al., 2021, Coleman-Cowger et al., 2018), potentially due to shared pathophysiology with growth abnormalities. Findings for preterm birth were mixed, with nearly half of studies reporting elevated risk and the other half reporting no significant association. Notably, there was no evidence linking co-exposure to elevated risk of NICU admissions or altered APGAR scores (Chabarria et al., 2016, Coleman-Cowger et al., 2018, Dunn et al., 2023, Gray et al., 2010, Shi et al., 2021, Waddell et al., 2024, Warshak et al., 2015). Overall, the strength of evidence suggesting a negative impact of prenatal co-exposure on neonatal outcomes gradually increased from respiratory distress syndrome, preterm birth, birth defects and physical development at the highest, whereas NICU stays and APGAR scores may be more reflective of the neonate’s immediate postnatal environment such as delivery complications. For behavioral outcomes, there was some evidence that prenatal co-exposure was associated with greater internalizing and externalizing symptomatology and emotion regulation/reactivity compared to single substance exposure (Eiden et al., 2018; Nadler et al., 2025, Stroud et al., 2018). In contrast, several studies reported that the effects of prenatal co-exposure were comparable to those of single-substance exposure (Lallai et al., 2022) or were no different than unexposed children (Eiden et al., 2018, Godleski et al., 2016, Godleski et al., 2018, Nadler et al., 2025, Perry et al., 2024, Schuetze et al., 2018). However, consumption patterns, rather than just mere exposure, may significantly influence the interaction between prenatal cannabis and NTP on behavioral outcomes; however, this was only investigated in one study (Nadler et al., 2025). Thus, future research should systematically assess patterns of use to better elucidate dose-response relationships between prenatal cannabis and NTP exposure and childhood behavior.

The eight cohort studies examining the association between prenatal co-exposure and cognitive outcomes found no interaction between prenatal cannabis and NTP exposure on any tests examined (Fried et al., 1992, Fried and Watkinson, 1988, Fried and Watkinson, 1990, Fried and Watkinson, 2000, Fried et al., 1992, Richardson et al., 1995, Willford et al., 2010). However, among non-cohort studies, effects of co-exposure on cognition emerged. A preclinical study demonstrated that in adolescent males, prenatal co-exposure elicited a deficit in sensory gating (indexed by PPI) that was not observed with prenatal nicotine or THC exposure alone (Lallai et al., 2022), suggesting that additive or synergistic effects between the substances underlie this enhanced effect. Additionally, a study in infants reported that the impact of co-exposure on attention may be ~40 % greater than that due to tobacco exposure alone (Stroud et al., 2018). Given that attention can modulate PPI (Filion et al., 1993), co-exposure effects may be most robust in the cognitive domain of attention. While some of the cohort studies reviewed did investigate attention (e.g., (Fried and Watkinson, 1988, Fried and Watkinson, 2000, Fried et al., 1992), co-exposure effects may have been statistically diluted in these studies given the heterogeneous samples.

Eight studies examined physiological outcomes and revealed a consistent pattern across select subdomains: prenatal co-exposure was associated with more adverse physiological effects compared to single-substance exposure and no exposure. Specifically, co-exposure was associated with lower cortisol levels and reduced cortisol reactivity in kindergarten-aged children (Eiden et al., 2020, Stroud et al., 2020), suggesting potential dysregulation of the HPA axis and impaired stress regulation. Similar patterns were observed with increased lethargy in infants (Stroud et al., 2018), higher obesity rates in middle childhood (Kong et al., 2023), and respiratory sinus arrhythmia in younger (Eiden et al., 2018) but not older toddlers (Schuetze et al., 2019). Evidence in the physiological domain is consistent and encouraging but requires replication to confirm these effects as studies investigating each subdomain are scarce.

**Fig. 2:**
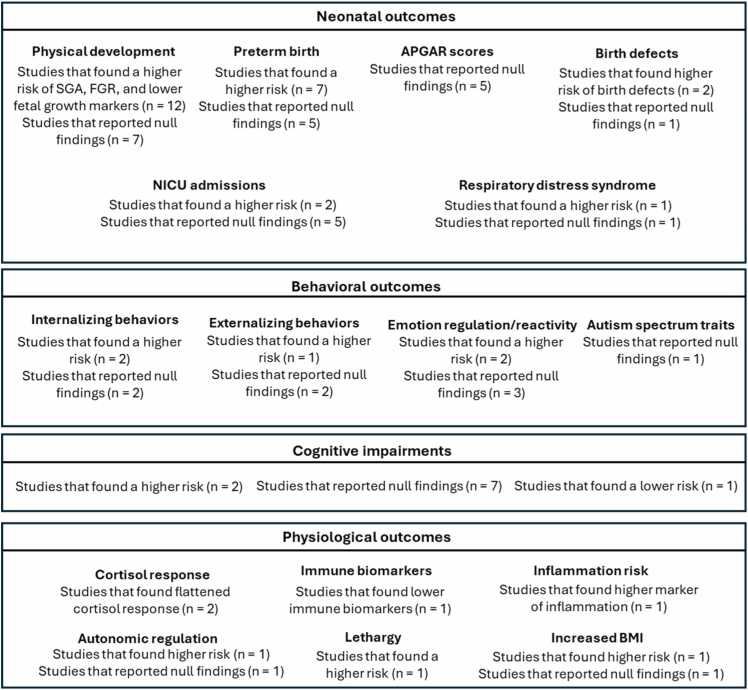
Summary of evidence comparing co-exposure to single- or no-substance exposure across neonatal, behavioral, cognitive, and physiological domains Abbreviations: APGAR, appearance, pulse, grimace, activity, and respiration; BMI, Body Mass Index; FGR, fetal growth restriction; NICU, neonatal intensive care unit; SGA, small for gestational age.

In sum, evidence suggests that select neonatal (i.e., physical development, birth defects, preterm birth), behavioral (i.e., internalizing behavior, emotion regulation), and physiological (i.e., immune and inflammation markers, cortisol response, anthropometrics) outcomes may reflect additive or synergistic effects of prenatal cannabis and NTP exposure, while support for cognitive effects remains limited. Fig. 2 provides a structured summary of the evidence across neonatal, behavioral, cognitive, and physiological outcomes comparing prenatal co-exposure to single-substance exposure and no exposure.

### 4.2 Potential mechanisms underlying co-exposure effects

Given the emerging association between prenatal co-exposure and offspring outcomes, we explored putative biological mechanisms, particularly those involving pharmacokinetic and pharmacodynamic pathways, that may underlie these effects.

Current evidence does not strongly support a pharmacokinetic interaction between nicotine and THC that amplifies their individual effects during prenatal exposure. For example, a 2016 review concluded that the metabolism of THC and nicotine are largely independent, with minimal evidence of enzyme inhibition or induction between the two substances (Anderson and Chan, 2016). However, a recent preclinical study reported that prenatal co-exposure resulted in lower plasma concentrations of both THC and nicotine compared to individual administration (Breit et al., 2022). While this indicates possible pharmacokinetic interactions, these are unlikely to explain the heightened adverse outcomes associated with co-exposure. Thus, pharmacokinetic mechanisms are unlikely to account for the observed effects of prenatal co-exposure on neonatal, behavioral or physiological outcomes in offspring.

A growing body of evidence suggests that the interactive effects of prenatal cannabis and NTP co-exposure may be mediated via pharmacodynamic mechanisms by activating cannabinoid and nicotinic receptors that are present in utero early in development. Research demonstrates that both cannabinoid and nicotinic receptors are expressed in the placenta as early as the first trimester (Banerjee et al., 2022, Rokeby et al., 2023). The binding of cannabis and NTP to their respective receptors has been shown to disrupt placental development and function (Holloway et al., 2014, Maia et al., 2019) by inducing oxidative stress in placental tissues, damaging placental cells and blood vessels (Banerjee et al., 2022). This damage impairs nutrient and oxygen exchange, resulting in placental insufficiency and compromised fetomaternal circulation (Banerjee et al., 2022). Such insufficiency has been linked to a range of developmental consequences, including neonatal outcomes (fetal growth restrictions, low birth weight, preterm birth), behavioral impairments (e.g., emotion regulation) as well as physiological dysregulation such as increased risk of obesity (Gagnon, 2003, John, 2022, Mohan et al., 2018, Preston et al., 2024).

Prenatal exposure to cannabis and NTP may also interfere with key neurotransmitter systems that are critical for intact neurodevelopment. Prenatal cannabis exposure has been shown to dysregulate dopaminergic, glutamatergic, GABAergic, serotonergic, and opioid signaling, particularly within corticolimbic circuits (Jenkins et al., 2025). Similarly, early activation of nicotinic acetylcholine receptors by prenatal NTP exposure can interfere with the maturation of these same circuits, altering neuronal excitability, receptor expression, and synaptic organization (Little et al., 2021). These neurochemical disruptions may compromise the structural and functional development of brain regions contributing to neonatal abnormalities, behavioral dysregulation, and long-term physiological impairments in co-exposed offspring.

Notably, both cannabis and NTP impact the development of the HPA axis (Micale and Drago, 2018, Rohleder and Kirschbaum, 2006), critical for regulating intrauterine homeostasis, maturation of vital organ systems, and ensuring the proper timing and sequence of fetal growth (Challis et al., 2001). While nicotine exposure has been shown to inhibit functional HPA development (Liu et al., 2012), prenatal cannabis exposure appears to remodel fetal HPA-axis development via disruptions of endocannabinoid-glucocorticoid signaling pathways (Franks et al., 2020). Early dysregulation of the HPA axis has been associated with increased risk of adverse neonatal outcomes (e.g., low birth weight, preterm birth, small for gestational age), behavioral outcomes, and physiological outcomes (e.g., higher body weight, increased risk of obesity) (Duthie and Reynolds, 2013, Schäffer et al., 2009, Wells and Lotfipour, 2023).

Evidence on the combined effects of prenatal cannabis and NTP exposure on placental maturation, neurotransmitter systems, and HPA axis development remains limited. However, given the well documented effects of each substance independently, it is plausible that co-exposure produces synergistic disruptions, exacerbating neonatal abnormalities, behavioral disturbances, and physiological dysregulation beyond the effects of either substance alone. While we highlight three plausible biological mechanisms, other pathways are likely involved and warrant investigation.

Lastly, it is possible that the observed co-exposure effects reflect pre-existing trait differences between mothers who co-use relative to those who only use one substance, rather than the effects of co-exposure itself on offspring outcomes. These trait differences, such as specific demographic factors (e.g., low socioeconomic status) and health-related behaviors (e.g., greater psychopathology) may independently predict increased risk for adverse outcomes in offspring (Coleman-Cowger et al., 2018).

### 4.3 Limitations

Several limitations in the studies included in this review may have contributed to the variability in findings.

First, many of the reviewed studies lacked one of the single exposure groups, which makes it difficult to isolate the specific contribution of each substance to co-use effects. Second, many of the included studies were derived from the same cohort, increasing the risk of systematic biases related to study design, data collection methods, and population characteristics, which may have influenced the outcomes. Further, there were only three animal studies included in our review. Preclinical studies are essential given their rigorous control over confounding factors that are common in human research (e.g., polysubstance use, environmental influences). Moreover, they play a crucial role in identifying the mechanisms underlying the adverse effects of prenatal substance exposure on offspring.

Third, in nearly half of the included studies, substance use was determined by maternal self-report (Bandoli et al., 2021, Brink et al., 2022, Chabarria et al., 2016, Crosland et al., 2024, Dunn et al., 2023, Fried and O′Connell, 1987, Fried et al., 1992, Fried and Watkinson, 1988, Fried and Watkinson, 1990, Fried and Watkinson, 2000, Fried et al., 1992, Garrison-Desany et al., 2022, Haight et al., 2021, Leemaqz et al., 2016, Nawa et al., 2020, Nguyen and Harley, 2022, Richardson et al., 1995, Shi et al., 2021, Sturrock et al., 2020, Willford et al., 2010). This method has inherent limitations as retrospective reporting can lead to underreporting or underestimation of substance use (Campbell et al., 2024, González-Colmenero et al., 2021). Further, since substance use during pregnancy is not recommended, substance use may have been underreported for fear of stigma, shame, or potential repercussions associated with disclosure (Garg et al., 2016). Thus, some infants classified as unexposed may have been exposed and may have affected results. Supporting this, studies found varying results depending on whether substance use was classified via self-report or biochemical verification (Abdelwahab et al., 2022, Gray et al., 2010), underscoring the need for objective measures to corroborate self-report substance exposure.

Fourth, prenatal substance exposure was often defined as a binary variable. This approach fails to account for variations in cannabis and NTP consumption patterns, which may influence outcomes in a dose-dependent manner, as was demonstrated with neonatal (Haight et al., 2021) and behavioral (Nadler et al., 2025) outcomes. Further, no study considered cannabis potency or strain/composition. Given the rising potency of cannabis (ElSohly et al., 2021), findings from older studies (Fried et al., 1992, Fried and Watkinson, 1988, Fried and Watkinson, 1990, Fried and Watkinson, 2000, Fried et al., 1992), may not fully capture the effects associated with present-day prenatal cannabis exposure. Similarly, with NTP prenatal exposure, outcomes may vary depending on whether nicotine was consumed via electronic cigarettes or tobacco via traditional combustible tobacco cigarettes. Therefore, future studies should better characterize substance use during pregnancy with respect to product type (e.g., e-cigarettes, smokeless tobacco), dose, potency, and route of administration.

Fifth, there was a lack of long-term follow-up studies. Most studies in this review focused on neonatal and early childhood outcomes, leaving a critical gap in understanding the trajectory of these effects over middle childhood and adolescence. Additional studies are needed to examine the effects of prenatal substance exposure during adolescence, a critical period when many neurodevelopmental disorders emerge, in order to fully understand its impact (Paus et al., 2008).

Lastly, we acknowledge a limitation of the review process itself which is subject to publication bias and may have skewed results.

### 4.4 Conclusions and future directions

This is the first review to identify the adverse consequences associated with prenatal cannabis and NTP co-exposure on neonatal, behavioral, cognitive, and physiological outcomes. These findings are consistent with previous reviews on the individual effects of prenatal cannabis and NTP exposure (Ernst et al., 2001, Sorkhou et al., 2024), but highlight the additive and potentially synergistic effects of co-using both substances. These results underscore the urgent need for targeted prevention strategies as rates of cannabis and NTP co-use, along with cannabis potency, continue to rise. Future research should further investigate the biological framework possibly underlying additive and synergistic effects on outcomes, while also identifying effective clinical interventions to mitigate risks to offspring. Moving forward, studies should incorporate appropriate comparison samples for exposure, validate substance use through biochemical assessments, and more thoroughly characterize exposure based on consumption patterns and cannabis potency.

## CRediT authorship contribution statement

**Mathilde Argote:** Writing – review & editing, Writing – original draft, Formal analysis. **Maryam Sorkhou:** Writing – review & editing. **Leah Hilson:** Writing – original draft, Formal analysis. **Rabin Rachel:** Writing – review & editing, Supervision, Methodology, Conceptualization.

## Funding

This work was supported by a 10.13039/501100000024Canadian Institutes of Health Research (Project grant 496033) to RAR and Fonds de Recherche du Quebec—Santé to RAR (297124).

## Declaration of Competing Interest

The authors declare that they have no known competing financial interests or personal relationships that could have appeared to influence the work reported in this paper.

## Footnotes

Appendix A Supplementary data associated with this article can be found in the online version at doi:10.1016/j.dadr.2025.100376.
