# Supplementary material for: A systematic review investigating prenatal cannabis and tobacco co-exposure: Impacts on neonatal, behavioral, cognitive and physiological outcomes
Source: Drug Alcohol Depend Rep. 2025 Aug 29;17:100376. doi: 10.1016/j.dadr.2025.100376 (PMC12451275; doi:10.1016/j.dadr.2025.100376)
Supplement: Supplementary file 1 — Supplementary material [file mmc1.docx]

Supplementary Table 1. Documentation of Search Strategy MEDLINE(R) ALL 1946 to May 09, 2025 (OVID)

| **1** | **Cannabis search terms** | Cannabis/ or Cannabinoids/ or Marijuana Smoking/ or Marijuana Abuse/ or "marijuana use".mp. or "marijuana use"/ or marijuana smoking/ or (THC or cannabis or cannabinoid* or marijuana).mp. | 74819 |
| --- | --- | --- | --- |
| **2** | **Nicotine search terms** | Nicotine/ or Electronic Nicotine Delivery Systems/ or Nicotine Chewing Gum/ or Nicotine Replacement Therapy/ or Tobacco Smoking/ or "Tobacco Use"/ or "Tobacco Use Disorder"/ or Tobacco, Smokeless/ or cigar smoking/ or cigarette smoking/ or nicotine.mp. or tobacco.mp. | 188911 |
| **3** | **Methods of administration** | smoking/ or pipe smoking/ or smoking reduction/ or smoking, non-tobacco products/ or tobacco smoking/ or vaping/ | 158111 |
| **4** | **Pregnancy/prenatal search terms** | Pregnancy/ or Pregnancy Outcome/ or Pregnant People/ or Prenatal Exposure Delayed Effects/ or Maternal Exposure/ or Abnormalities, Drug-Induced/ or Fetal Development/ or (pregnan* or prenatal* or pre-natal* or fetal* or baby or babies or neonat* or perinatal* or peri natal*).mp. | 1680689 |
| **5** |  | 1 and (2 or 3) and 4 | 899 |

Supplementary Table 2. Documentation of Search Strategy Embase 1996 to 2025 Week 19 (OVID)

| **1** | **Cannabis search terms** | Cannabis/ or Cannabinoids/ or Marijuana Smoking/ or Marijuana Abuse/ or "marijuana use".mp. or "marijuana use"/ or marijuana smoking/ or (THC or cannabis or cannabinoid* or marijuana).mp. | 106216 |
| --- | --- | --- | --- |
| **2** | **Nicotine search terms** | Nicotine/ or Electronic Nicotine Delivery Systems/ or Nicotine Chewing Gum/ or Nicotine Replacement Therapy/ or Tobacco Smoking/ or "Tobacco Use"/ or "Tobacco Use Disorder"/ or Tobacco, Smokeless/ or cigar smoking/ or cigarette smoking/ or nicotine.mp. or tobacco.mp. | 585988 |
| **3** | **Methods of administration** | smoking/ or pipe smoking/ or smoking reduction/ or smoking, non-tobacco products/ or tobacco smoking/ or vaping/ | 397880 |
| **4** | **Pregnancy/prenatal search terms** | Pregnancy/ or Pregnancy Outcome/ or Pregnant People/ or Prenatal Exposure Delayed Effects/ or Maternal Exposure/ or Abnormalities, Drug-Induced/ or Fetal Development/ or (pregnan* or prenatal* or pre-natal* or fetal* or baby or babies or neonat* or perinatal* or peri natal*).mp. | 1474587 |
| **5** |  | 1 and (2 or 3) and | 1850 |

Supplementary Table 3. Documentation of Search Strategy PsycINFO 1967 May 2025 Week 1 (OVID)

| **1** | **Cannabis search terms** | Cannabis/ or Cannabinoids/ or Marijuana Smoking/ or Marijuana Abuse/ or "marijuana use".mp. or "marijuana use"/ or marijuana smoking/ or (THC or cannabis or cannabinoid* or marijuana).mp. | 34538 |
| --- | --- | --- | --- |
| **2** | **Nicotine search terms** | Nicotine/ or Electronic Nicotine Delivery Systems/ or Nicotine Chewing Gum/ or Nicotine Replacement Therapy/ or Tobacco Smoking/ or "Tobacco Use"/ or "Tobacco Use Disorder"/ or Tobacco, Smokeless/ or cigar smoking/ or cigarette smoking/ or nicotine.mp. or tobacco.mp. | 62658 |
| **3** | **Methods of administration** | smoking/ or pipe smoking/ or smoking reduction/ or smoking, non-tobacco products/ or tobacco smoking/ or vaping/ | 39202 |
| **4** | **Pregnancy/prenatal search terms** | Pregnancy/ or Pregnancy Outcome/ or Pregnant People/ or Prenatal Exposure Delayed Effects/ or Maternal Exposure/ or Abnormalities, Drug-Induced/ or Fetal Development/ or (pregnan* or prenatal* or pre-natal* or fetal* or baby or babies or neonat* or perinatal* or peri natal*).mp. | 130398 |
| **5** |  | 1 and (2 or 3) and 4 | 468 |

Supplementary Table 4. Critical Appraisal Results – Quality Evaluation of Included Studies using Johanna Briggs Institute Checklist for Cohort Studies

|  | Johanna Briggs Institute Checklist for Cohort Studies | | | | | | | | | | |  |
| --- | --- | --- | --- | --- | --- | --- | --- | --- | --- | --- | --- | --- |
|  | **1** | **2** | **3** | **4** | **5** | **6** | **7** | **8** | **9** | **10** | **11** | **Score out of 10*** |
| Abdelwahab et al., 2022 | Y | Y | Y | Y | Y | N/A | Y | Y | Y | Y | Y | 10 |
| Bandoli et al., 2021 | Y | Y | U | Y | Y | N/A | Y | Y | Y | N | Y | 8 |
| Brink et al., 2022 | Y | Y | U | Y | Y | N/A | Y | Y | Y | N | Y | 8 |
| Chabarria et al., 2016 | Y | U | U | Y | Y | N/A | Y | Y | Y | N | Y | 7 |
| Coleman-Cowger et al., 2018 | Y | Y | Y | Y | Y | N/A | Y | Y | Y | N | Y | 9 |
| Crosland et al., 2024 | Y | Y | U | Y | Y | N/A | Y | Y | Y | N | Y | 8 |
| Dunn et al., 2023 | Y | Y | U | Y | Y | N/A | Y | Y | Y | N | Y | 8 |
| Fried & O'Connell, 1987 | U | Y | U | Y | Y | N/A | U | Y | Y | N | U | 5 |
| Gray et al., 2010 | U | Y | Y | N | Y | N/A | Y | Y | Y | N | Y | 7 |
| Haight et al., 2021 | Y | Y | U | Y | Y | N/A | Y | Y | Y | N | Y | 8 |
| Kelm et al., 2024 | Y | Y | Y | Y | Y | N/A | Y | Y | Y | Y | Y | 10 |
| Leemaqz et al., 2016 | Y | Y | N | Y | Y | N/A | Y | Y | Y | Y | Y | 9 |
| Nawa et al., 2020 | Y | Y | N | Y | Y | N/A | Y | Y | Y | N | Y | 8 |
| Nguyen & Harley, 2022 | Y | Y | U | Y | Y | N/A | Y | Y | Y | N | Y | 8 |
| Nutor et al., 2024 | Y | Y | N | Y | Y | N/A | Y | Y | Y | N | Y | 8 |
| Shi et al., 2021 | Y | Y | U | Y | Y | N/A | Y | Y | Y | N | Y | 8 |
| Sturrock et al., 2020 | Y | Y | U | N | N | N/A | Y | Y | Y | N | Y | 6 |
| Waddell et al., 2023 | Y | U | U | Y | Y | N/A | Y | Y | U | N | Y | 6 |
| Warshak et al., 2015 | Y | N | Y | Y | Y | N/A | Y | Y | Y | N | Y | 8 |
| Eiden, Schuetze, et al., 2018 | Y | Y | Y | Y | Y | N/A | Y | Y | Y | N | Y | 9 |
| Eiden, Zhao, et al., 2018 | Y | Y | Y | Y | Y | N/A | Y | Y | Y | N | Y | 9 |
| Garrison-Desany et al., 2022 | Y | Y | U | Y | Y | N/A | Y | Y | Y | Y | Y | 9 |
| Godleski et al., 2016 | Y | Y | Y | Y | Y | N/A | Y | Y | U | U | Y | 8 |
| Godleski et al., 2018 | Y | Y | Y | Y | Y | N/A | Y | Y | U | N | Y | 8 |
| Nadler et al., 2024 | Y | Y | N | Y | Y | N/A | Y | Y | N | N | Y | 7 |
| Perry et al., 2024 | Y | Y | Y | Y | Y | N/A | Y | Y | Y | Y | Y | 10 |
| Schuetze et al., 2018 | Y | Y | Y | Y | Y | N/A | Y | Y | Y | Y | Y | 10 |
| Schuetze et al., 2019 | Y | Y | Y | Y | Y | N/A | Y | Y | Y | Y | Y | 10 |
| Stroud et al., 2018 | Y | Y | Y | Y | Y | N/A | Y | Y | Y | N | Y | 9 |
| Fried, O'Connell, et al., 1992 | U | Y | U | Y | Y | N/A | Y | Y | Y | N | Y | 7 |
| Fried & Watkinson, 1988 | U | Y | U | Y | Y | N/A | Y | Y | Y | N | Y | 7 |
| Fried & Watkinson, 1990 | U | Y | U | Y | Y | N/A | Y | Y | Y | N | Y | 7 |
| Fried & Watkinson, 2000 | U | Y | U | Y | Y | N/A | Y | Y | Y | N | Y | 7 |
| Fried, Watkinson, et al., 1992 | U | Y | U | Y | Y | N/A | Y | Y | Y | N | Y | 7 |
| Richardson et al., 1995 | Y | Y | U | Y | Y | N/A | U | Y | Y | N | Y | 7 |
| Shisler et al., 2024 | U | Y | Y | Y | Y | N/A | Y | Y | Y | Y | Y | 9 |
| Willford et al., 2010 | Y | Y | U | Y | Y | N/A | Y | Y | Y | N | Y | 8 |
| Eiden et al., 2020 | Y | U | Y | Y | Y | N/A | Y | Y | Y | N | Y | 8 |
| Molnar et al., 2018 | Y | Y | Y | U | N | N/A | Y | Y | Y | N | Y | 7 |
| Simon et al., 2023 | Y | Y | Y | Y | Y | N/A | Y | Y | Y | N | Y | 9 |
| Stroud et al., 2020 | Y | Y | Y | Y | Y | N/A | Y | Y | Y | N | Y | 9 |
| Kong et al., 2023 | Y | Y | Y | Y | Y | N/A | Y | Y | Y | Y | Y | 10 |

Y: Yes = 1; N: No = 0; U: Unclear = 0; N/A = Not applicable.

*Score out of a possible 10, as question 6 is irrelevant to the studies examined.

Criteria: 1. Were the two groups similar and recruited from the same population? 2. Were the exposures measured similarly to assign people to both exposed and unexposed groups? 3. Was the exposure measured in a valid and reliable way? 4. Were confounding factors identified? 5. Were strategies to deal with confounding factors stated? 6. Were the groups/participants free of the outcome at the start of the study (or at the moment of exposure)? 7. Were the outcomes measured in a valid and reliable way? 8. Was the follow up time reported and sufficient to be long enough for outcomes to occur? 9. Was follow up complete, and if not, were the reasons to loss to follow up described and explored? 10. Were strategies to address incomplete follow up utilized? 11. Was appropriate statistical analysis used?

Supplementary Table 5. Critical Appraisal Results – Quality Evaluation of Included Preclinical Studies using Johanna Briggs Institute Checklist for Randomized Control Trials

|  | Johanna Briggs Institute Checklist for Cohort Studies | | | | | | | | | | | | |  | |
| --- | --- | --- | --- | --- | --- | --- | --- | --- | --- | --- | --- | --- | --- | --- | --- |
|  | **1** | **2** | **3** | **4** | **5** | **6** | **7** | **8** | **9** | **10** | **11** | **12** | **13** | **Score out of 11** |  |
| Breit et al., 2022 | Y | U | Y | N/A | U | Y | Y | Y | Y | Y | N/A | Y | Y | 9 |  |
| Hussain et al., 2022 | U | U | Y | N/A | U | Y | Y | Y | Y | U | N/A | Y | Y | 7 |  |
| Lallai et al., 2022 | U | U | Y | N/A | U | Y | Y | Y | Y | Y | N/A | Y | Y | 8 |  |

Y: Yes = 1; N: No = 0; U: Unclear = 0; N/A = Not applicable.

*Score out of a possible 11, as questions 4 and 11 are irrelevant to the studies examined.

Criteria: 1. Was true randomization used for assignment of participants to treatment groups? 2. Was allocation to treatment groups concealed? 3. Were treatment groups similar at the baseline? 4. Were participants blind to treatment assignment? 5. Were those delivering the treatment blind to treatment assignment? 6. Were treatment groups treated identically other than the intervention of interest? 7. Were outcome assessors blind to treatment assignment? 8. Were outcomes measured in the same way for treatment groups? 9. Were outcomes measured in a reliable way? 10. Was follow up complete and if not, were differences between groups in terms of their follow up adequately described and analysed? 11. Were participants analysed in the groups to which they were randomized? 12. Was appropriate statistical analysis used? 13. Was the trial design appropriate and any deviations from the standard RCT design (individual randomization, parallel groups) accounted for in the conduct and analysis of the trial?
